# Purchasing Habits, Sustainability Perceptions, and Welfare Concerns of Italian Consumers Regarding Rabbit Meat

**DOI:** 10.3390/foods11091205

**Published:** 2022-04-21

**Authors:** Stefania Crovato, Anna Pinto, Guido Di Martino, Giulia Mascarello, Valentina Rizzoli, Silvia Marcolin, Licia Ravarotto

**Affiliations:** 1 Istituto Zooprofilattico Sperimentale delle Venezie, 35020 Legnaro, Italy; scrovato@izsvenezie.it (S.C.); gdimartino@izsvenezie.it (G.D.M.); gmascarello@izsvenezie.it (G.M.); smarcolin@izsvenezie.it (S.M.); lravarotto@izsvenezie.it (L.R.); 2 Department of Communication and Social Research, Sapienza University of Rome, 00198 Rome, Italy; valentina.rizzoli@uniroma1.it

**Keywords:** purchasing habits, consumer perceptions, animal welfare, Italian consumers

## Abstract

Several factors drive consumer behavior in buying meat, particularly rabbit meat. The consumption of rabbits has decreased in Europe, and the main causes of this trend are an increasing association with the perception of rabbits as pets, consumers’ changes in lifestyle, and eating habits. Additionally, increasing attention is paid to ethical issues regarding animal welfare. Investigating consumers’ knowledge, perceptions, and concerns about rabbit meat production and consumption are crucial for improving market strategies. This study investigated consumers’ perspectives of rabbit meat to provide useful information to producers for promoting this sector to consumers. A mixed-methods research design was applied. Qualitative data were collected through four focus group discussions involving 32 consumers and quantitative data through a semi-structured questionnaire administered nationally and completed by 1001 consumers. The limited use of antibiotics in inbreeding and the absence of animal suffering are factors that most influence consumers’ willingness to purchase rabbit meat. Rural/domestic farms were recognized as places where animal welfare measures can be applied and have a positive influence not only on organoleptic quality but also on ethical value and food safety. The cage was perceived as unsuitable for rabbit growth and was oriented to a process of fattening based on industrial feed and antibiotics. Respondents sought information on rabbit farming during purchase, and the label was the most used tool. The rabbit meat production sector should consider these results to meet consumers’ demands and raise awareness among operators on the use of animal welfare-based farming systems, helping to build a more positive image of the rabbit meat industry.

## 1. Introduction

Understanding consumers’ purchasing behaviors and eating habits are fundamental to supporting and promoting different food chains. There are complex and heterogeneous factors that drive consumer behavior in buying a specific kind of meat, and they cannot be generalized to other meats. Studies on market segmentation reveal that each group of consumers has distinct needs, perceptions, and knowledge toward a specific product, especially meat [1]. The analysis of the various consumer features and behaviors is also relevant for developing targeted marketing strategies. It can be relevant for the successful promotion of the purchase and consumption of rabbit meat, which is currently limited to only a few major producers (Italy, Spain, and France) contributing to most of the total production [2]. On the other hand, meat rabbits are the second most farmed species in the EU regarding the number of animals, with the majority reared in cages with inadequate welfare standards [3]. Italy is among the top five rabbit meat producers in the world, and its national production is increasing [4]. However, rabbit meat consumption has decreased in the last five years in the Mediterranean region and Italy [5,6], especially among the younger population [7]. Studies show that the main causes of this trend are an increasing association with the perception of rabbits as pets, a consumer’s change in lifestyle and eating habits (i.e., the time spent cooking and eating being reduced and the ease of food preparation being a key aspect), and increasing attention to ethical issues regarding animal welfare [8,9]. Consumers are interested in animal housing and welfare standards. Citizens have recently asked the EU Parliament for a pronouncement against the use of cages in livestock, with the resolution, ‘End the cage age’, which received 1.4 million validated signatures in the EU [3]. Some studies show that concern for animal welfare is a key point in consumers’ meat choices, as well as the most common reason for meat consumption reduction [9,10]. An increasing number of people are sensitive to the suffering and slaughtering of farm animals, choosing to stop eating meat [11].

Currently, for the meat rabbit sector, different types of housing systems are available in the market, ranging from conventional cages (called ‘bicellular’, i.e., barren cages for two growing rabbits) to enriched cages (i.e., containing one litter and characterized by more space, a raised platform, environmental enrichments, and a plastic footrest) and ‘park systems’ (i.e., an indoor area for keeping different litters together, containing a raised platform, environmental enrichments, and a plastic footrest) [12]. Enriched and alternative systems are still not widely used in the field, although they have proven to be non-penalizing and economically sustainable [13]. However, the level of consumer knowledge regarding the housing system in the meat rabbit sector is unclear. Therefore, the investigation of consumers’ knowledge, perceptions, and concerns about meat rabbit production is of utmost importance to improve market strategies, develop a positive economic effect for the market, and guarantee the survival of knowledge and technical expertise developed by breeders and producers in this sector.

This study presents data from an Italian research project concerning the meat rabbit sector, focusing on a comparison of conventional, enriched, and alternative housing systems from different perspectives (welfare assessment, economic impact, and consumer). This study was realized by the Istituto Zooprofilattico Sperimentale delle Venezie (IZSVe) and funded by the Italian Ministry of Health. The data presented in the text refer to social research on consumers’ purchasing behaviors and eating habits and their perception of breeding methods, food safety, and animal welfare in the rabbit sector.

This study aims to provide useful information to producers to implement focused marketing strategies oriented toward the promotion of rabbit meat consumption among consumers, improve the production chain of this sector, and raise awareness among operators regarding the use of animal welfare-based farming systems.

## 2. Materials and Methods

### 2.1. Quantitative and Qualitative Methods

This study adopted a mixed method design. Qualitative data were collected through focus group discussions [14] to deeply explore consumers’ social and ethical concerns associated with rabbit meat production and consumption. Four main aspects were investigated: consumption and purchase of rabbit meat, perception toward rabbit meat safety, and opinions regarding animal welfare in rabbit breeding farms. For consistency, the same mediator facilitated all focus groups. The content of each discussion was audio-recorded and then transcribed. Each focus group measured approximately 1.5 h in length.

Quantitative data were collected using a semi-structured questionnaire [15] administered at the national level using computer-assisted web interviewing (CAWI). Questions were developed based on the research team’s experience and the specific knowledge needs of the project. The questionnaire was divided into the following sections: (1) rabbit meat consumption and purchase; (2) knowledge and perception of rabbit breeding and animal welfare; and (3) socio-demographic characteristics of respondents. The questionnaire consisted of 22, mainly closed-ended, questions (with single or multiple choices) and Likert-scale; only four questions required an open answer and were analyzed as qualitative data.

Corresponding to the article’s objective, only some questions from the questionnaire (Table 1) were included in the analysis reported below.

Quantitative and qualitative data were collected and analyzed separately and then merged in the overall analysis and interpretation. The collected data were treated according to the General Data Protection Regulation (EU) 2016/679.

### 2.2. Participants

Meat consumers living in Italy were included in the study. Two groups of participants (total *n* = 32) were recruited for the qualitative phase through convenience sampling based on willingness to participate, age (younger ≤ 50 years; older > 50 years), and frequency of rabbit meat consumption (frequent and occasional consumers). The discussions were conducted in three cities (Padova, Vicenza, and Bologna) in northern Italy between December 2018 and January 2019.

In the national survey, participants were selected through quota sampling based on their gender, age, and geographical area. In total, 1001 consumers completed the questionnaire. The administration was conducted in January 2019 and was supported by a company specializing in market research and opinion polling.

To comply with the privacy policy, there was a privacy agreement request both to participants of the survey and focus group discussions. In the first case, the agreement was requested by a checkbox at the beginning of the online questionnaire; in the second case, a paper document was signed by participants before the focus groups began.

### 2.3. Analysis

Qualitative (focus group transcriptions and two open-ended questions from the questionnaire) and quantitative (questionnaire) data were initially analyzed separately and then compared to identify convergent and divergent findings [16].

#### 2.3.1. Qualitative Data

Data collected by focus groups and the two open questions included in the questionnaire were analyzed using automatic text analyses [17]. Three corpora were created. One contained all transcriptions of the four focus group discussions, and two contained all answers collected through each open-ended question: (1) ‘How do you think rabbits are raised on industrial farms?’ and (2) ‘For you, what is the meaning of the expression animal welfare?’ Lexicometric measures (Table 2) were calculated to evaluate lexical richness and the appropriateness of applying automatic text analysis (type/token ratio < 20%; hapax legomena < 50%). The measures can be considered acceptable, even if hapaxes exceed 50% because they are frequent with this type of text (speed writing and speech transcription).

First, using TaLTaC2 software (University of Rome, Rome, IT) [18,19], the corpora were pre-processed for automatic analysis. Uppercase letters have been replaced with lowercase and multi-words (meaningful sequences of words), according to the relative I.S. index [18]. After a manual check, frequencies more than 4 were individuated and considered a textual unit.

Second, the Reinert method [20] was applied to each corpus separately. This method, implemented in the IRaMuTeQ software (version 0.7, Pierre Ratinaud, Lerasse Laboratory, Toulouse, France) [21], consists of automated content analysis (i.e., the process of collecting, coding, analyzing, and interpreting the information present in one or more texts by returning its content in a new form [22]) and allows to quantitatively analyzing qualitative data systematically [23,24]. It was used to individuate clusters of words that refer to a common meaning [25], that is, topics, because they appear together (co-occur) in the same portion of text. Then, the association (according to the chi-square index) between each cluster and consumer characteristics (age and consumption frequency) was observed. Each cluster was explored in depth by observing the words associated with it in their context of use (i.e., the text). The interpretation of the results was carried out by three different authors (members of the research team) with the aim to agree and validate the findings.

#### 2.3.2. Quantitative Data

Questionnaire results were analyzed using univariate and bivariate statistical techniques [15]. Because the age of the respondents was one of the criteria used in the selection of focus group participants, this variable was used to identify any differences in the opinions and perceptions of the national survey participants owing to different ages. The *t*-test for independent samples was used to assess differences in the mean values of variables expressed on a 1–10 Likert scale between two groups of respondents: those aged 48 or under and those aged over 48. The choice to set the threshold at 48 years was derived from the average value obtained for the age of the respondents.

Fisher’s exact test was used to assess the differences in the distribution of categorical variables. Finally, a chi-square test was performed to investigate the dependent relationships between categorical variables.

Quantitative analyses were performed using Statistical Package for Social Science (SPSS) software (version 25.0.0.0) for Windows (SPSS Inc., Chicago, IL, USA). The level of statistical significance was set at 5% (α = 0.05).

## 3. Results

To best present the study results, the survey data were reported first and then those of the focus group discussions for more detailed insights.

### 3.1. Characteristics of Rabbit Meat Consumer

#### 3.1.1. Survey Results

**Figure 1:**
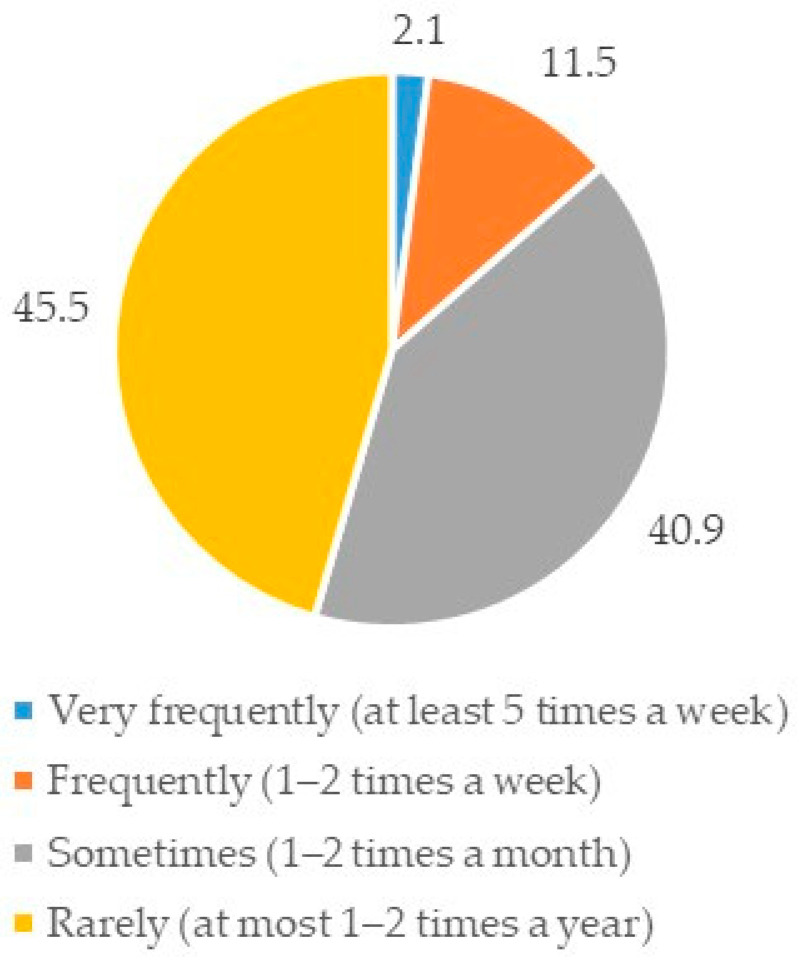
How often do you eat rabbit meat? (%, *n* = 705).

In total, 2334 consumers were invited to participate in the survey, and 1001 completed the questionnaire (response rate = 43%). Among the respondents, 70.4% (*n* = 705) declared that they would eat rabbit meat. This segment of the sample consisted mainly of ‘male’ consumers (51.8%), aged between ‘36 and 48 years’ (26%) with an ‘occupation’ (58.8%) and a ‘high school diploma’ (52.6%), living in ‘Northern Italy’ (45.4%) with a ‘partner and children’ (33.6%). No economic problems were reported by respondents (51.4%) (Table 3). This group of consumers ‘rarely’ ate rabbit meat, at ‘most 1–2 times during the year’ (45.5%) (Figure 1). Additionally, 71.4% (*n* = 503) reported that they purchased rabbit meat.

#### 3.1.2. Focus Group Results

Among the 32 participants in the focus group discussions, 20 were frequent consumers and 12 were occasional consumers (Table 4).

**Figure 2:**
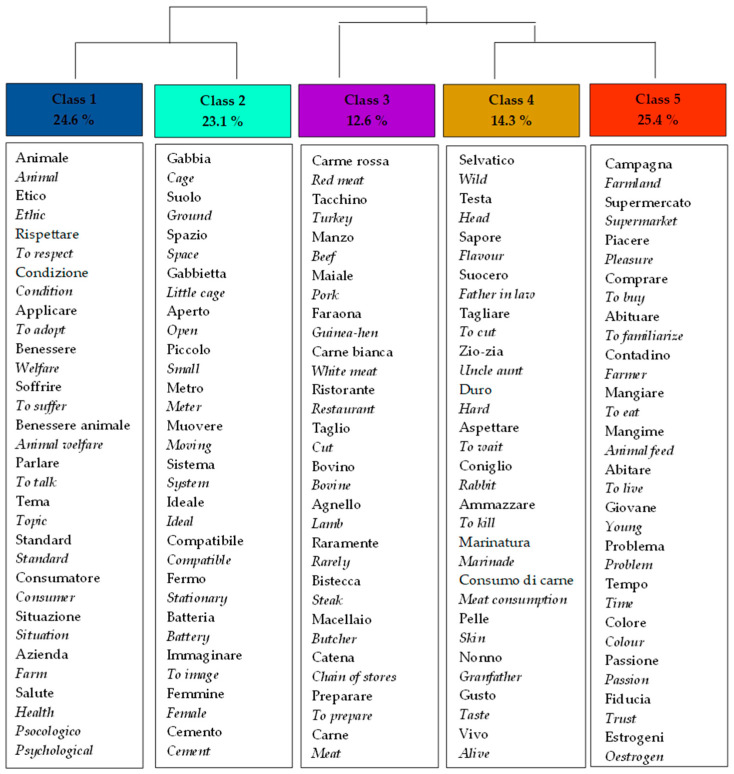
Five clusters of focus group discussions were identified by the Reinert method with the translation of words from Italian to English.

Five clusters (Figure 2) were identified from the corpus built based on focus group discussions. The clusters concern (1) animal welfare and ethical issues, (2) breeding methods, (3) differences between rabbit meat and other types of meat, (4) home preparation and consumption of rabbit meat, and (5) rabbit meat purchasing. These topics are discussed in depth in parallel with the presentation of the questionnaire results.

**Figure 3:**
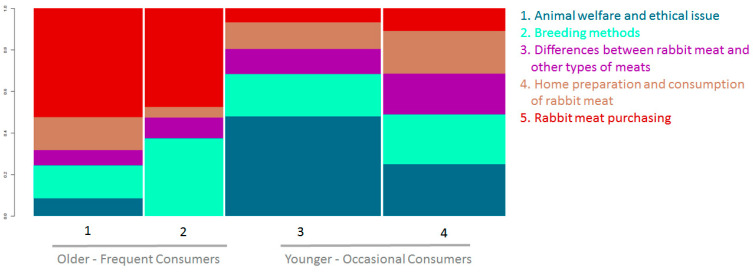
Distribution of the clusters identified among the four focus groups.

Older frequent consumers discussed the different farming methods (intensive vs. extensive) concerning their purchasing behavior for rabbit meat products. In contrast, younger-occasional consumers preferred to discuss the ethical issues of breeding and welfare linked to public health protection (Figure 3).

### 3.2. Purchasing Behaviors of Rabbit Meat

#### 3.2.1. Survey Results

In this paragraph, the reported data refer to respondents who declared both eating and purchasing rabbit meat (*n* = 503). The ‘supermarket’ was most frequently chosen by consumers for purchasing (60.4%), followed by the ‘butcher’s store’ (27.8%). Some respondents purchased rabbit meat ‘directly from the breeder’ (10.3%), and only a few respondents purchased rabbit meat from the ‘street market’ (1.2%) and ‘online’ (0.2%). Most samples preferred to ‘buy chopped rabbit’ (66.8%) rather than the ‘whole carcass’ (28.8%). Few respondents declared that they usually buy rabbit meat products that are ‘ready to cook’ (such as rabbit hamburgers or skewers) (3.8%) and already cooked ‘ready-to-eat meat’ (0.6%).

Rabbit meat consumers considered that ‘it has been bred with responsible use of antibiotics’ (m = 8.09), ‘it looks good’ (m = 7.92), and ‘it has been raised without suffering’ (m = 7.46) as the most important factors when purchasing rabbit meat, as shown in Table 5. There were no statistically significant differences in the mean values obtained for the investigated factors between those aged ≤ 48 years and those aged > 48 years.

**Figure 4:**
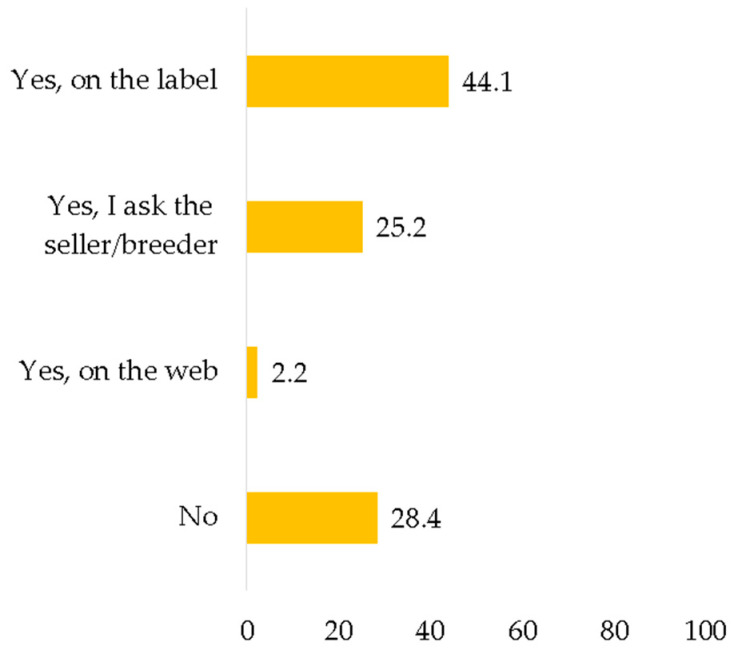
When you buy rabbit meat, have you ever looked for information on how the animal was raised? (%, *n* = 503).

71.5% of consumers declared to search for information on rabbit breeding methods during the meat purchase, particularly through the label, as shown in Figure 4.

#### 3.2.2. Focus Group Results

From the survey data, the cluster concerning the purchase of rabbit meat (cluster 5, 25.4% of classified text) showed that supermarkets and butcher’s stores are primarily where both younger and older consumers buy rabbit meat. The most frequent explanation of this choice concerned consumers’ trust in the food chain control system and the butcher with whom it is possible to have direct dialogue, as shown in the excerpt reported below.

*I assume that the controls perform well, so the product I find in the supermarket is safe (younger-occasional consumer).*

*You should trust when you go to the butcher or supermarket and hope to find a fair person (younger-occasional consumer).*

However, older rabbit meat consumers agreed that the taste of meat purchased directly from farms is better than that of rabbit meat that comes from controlled distribution.

*I usually buy [rabbit meat] from a friend of a mine, who is a breeder. I buy it occasionally at the supermarket, but the flavor is not the same (older-frequent consumer).*

In the focus group discussion, consumers noted a difference in taste between rabbits that came from large retailers, such as supermarkets, and those bought directly from farmers. Meat coming from ‘small farms’ or ‘domestic farms’ was defined as tastier and firmer. As reported below, different perceptions of rabbit meat emerged among older participants based on the farming methods used:

*On domestic farms, it takes six to eight months to raise an animal. An industry will go out of business with this timeline. When you eat an animal that is raised for an extended period, the bones remain attached to the meat, the taste is different, and it is better overall. Industries cannot achieve this. (older-frequent consumer)*

### 3.3. Perception and Knowledge of Animal Welfare in Rabbit Breeding

#### 3.3.1. Survey Results

**Figure 5:**
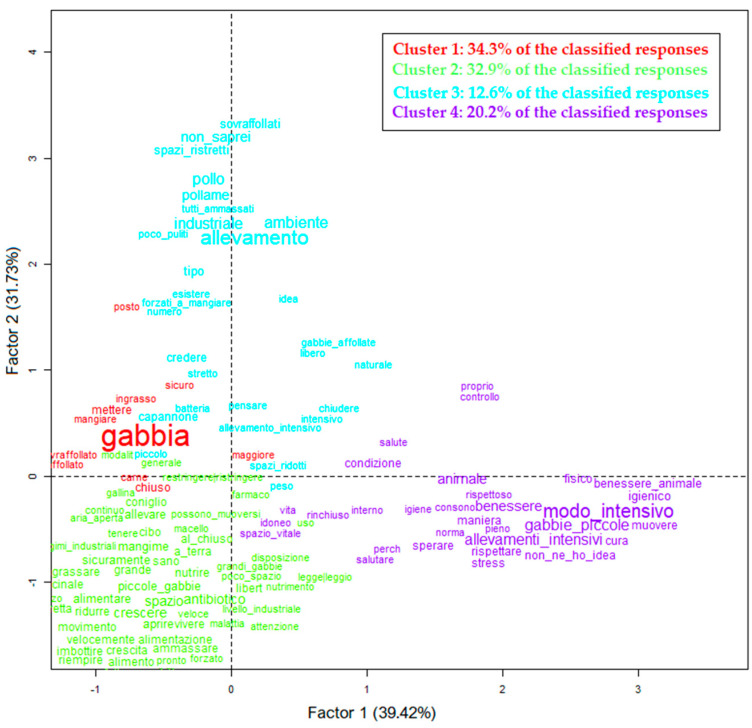
How do you think rabbits are raised on industrial farms? Write a short description below (*n* = 499). Projection of the four clusters identified (Reinert method) on the Cartesian plane. Each color represents a cluster. The size of the words is proportional to the association score (chi-square index) with the corresponding cluster. The translation of words is available in Appendix A, Figure A1.

Respondents (*n* = 1001) confirmed the results of the qualitative data reported in the previous paragraph. They agreed with the following statement: Meat from industrial farms is less tasty than meat from non-industrial farms (‘somewhat or strongly agree’ = 81.2%; ‘not at all or slightly agree’ = 18.8%). Consumers’ knowledge of breeding methods was investigated through the open question: ‘How do you think rabbits are raised on industrial farms? Write a short description below’. Of the respondents, 30.3% (*n* = 303) declared that they did not know how rabbits were raised. The answers given by the remaining respondents (*n* = 698) were analyzed using the Reinert method. As shown in Figure 5, 499 responses were classified, and five clusters were identified.

Cluster 1 (34.3%) identified the cage (*gabbia*) as the main method for rabbit breeding; Cluster 2 (32.9%) included words that describe and characterize the cage as small (*piccole*) and unsuitable for the growth and movement of the animal (*movimento, spazio, crescita*). ‘Cage’ has been mentioned as a method for fattening (*ingrasso*), associated with the use of industrial feed (*mangimi industriali*) and antibiotics (*antibiotici*). Cluster 3 (12.6%) showed similarities between rabbit and poultry industrial breeding methods. They were both defined as overcrowded (*sovraffollati, ammassati*) and unclean (*poco puliti*), where animals have force-feeding (*forzati a mangiare*). In Cluster 4 (20.2%), the topic of animal welfare (*benessere animale*) was mentioned. In particular, it has emerged that it is not possible to guarantee animal welfare in intensive breeding for several animal stress conditions (*stress*), mainly because of a lack of adequate living space (*spazio vitale*) and inappropriate hygienic conditions (*condizioni, igienico*).

**Figure 6:**
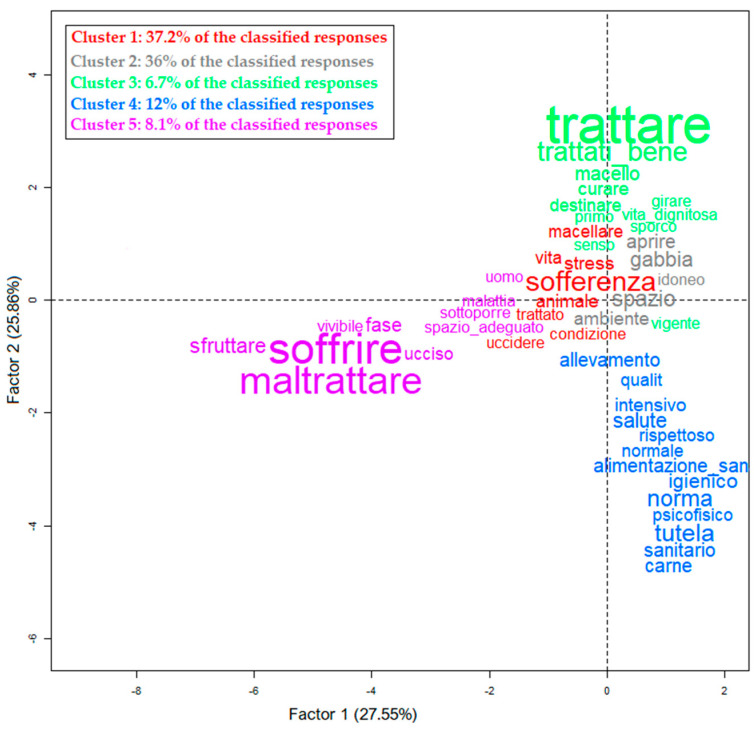
For you, what is the meaning of the expression animal welfare? Write your definition below (*n* = 792). Projection of the five clusters identified (Reinert method) on the Cartesian plane. Each color represents a cluster. The size of the words is proportional to the association score (chi-square index) with the corresponding cluster. The translation of words is available in Appendix B, Figure A2.

To the question, ‘Considering the rabbit industry, is the use of cages in breeding a method compatible with animal welfare?’ (*n* = 1.001), 64.6% of the sample said ‘no’. Only 6.8% stated ‘yes’, and 28.6% ‘did not know’. The perception of welfare animals was examined before providing the definition of animal welfare to all respondents (*n* = 1.001) with the open question, ‘For you, what is the meaning of the expression animal welfare? Write the definition below‘. The responses were analyzed using the Reinert method, which allowed the identify five clusters regarding the meaning of animal welfare (Figure 6). Of the respondents, 933 expressed their perspectives; only 792 responses were included in the cluster analysis, and 141 were found to be unclassifiable.

In Cluster 1 (37.2%), animal welfare was described as the absence of stress (*stress*) and suffering (*sofferenza*) of animals from birth to slaughter. Cluster 2 (36%) is concerned with the possibility of animals living in adequate spaces by ensuring the free movement of the animal (*spazio, libero, muovere*) and having healthy and appropriate feed (*alimentazione, mangiare,* and *sano*). Cluster 3 (6.7%) described positive and relevant aspects of animal welfare measures applied in the extensive farm: ensuring dignified living conditions (*condizioni dignitose*), caring for animals (*curare*), and treating animals with respect (*trattare bene*). Cluster 4 (12%) associated animal welfare with the regulation for protecting animals’ psychophysical conditions during breeding (*tutela, norma, legge,* *and* *psicofisico*). Cluster 5 (8.1%) was characterized by words referring to the absence of exploitation and suffering of farm animals (*soffrire, maltrattare,* and *sfruttare*). No significant associations between the five clusters and demographic characteristics emerged in Cluster 3. Only this cluster was associated with the group that did not consume rabbit meat (chi-square index 21.49, *p* < 0.001).

Respondents were asked a specific question concerning consumers’ knowledge of animal welfare legislation (‘In general, do you think there are regulations that protect animal welfare on farms?’). Of the sample, 65.3% declared ‘yes’, 12.8% ‘no’, and 21.9% chose ‘I don’t know’.

The level of consumer agreement with animal welfare statements was then investigated. Only 30.9% of the respondents who selected the options ‘somewhat agree’ or ‘strongly agree’ with the statement, ‘Industrial breeding facilities are careful to ensure animal welfare’ (‘not at all’ or ‘slightly agree’ = 69.1%). Alternatively, nearly all of the sample (84.1%) agreed with the sentence, ‘Only animals raised outdoors live according to animal welfare’ (‘not at all’ or ‘slightly agree’ = 15.9%).

#### 3.3.2. Focus Group Results

The topic of animal welfare was also deeply investigated during focus group discussions. The analysis of the portions of text concerning this topic showed agreement among all consumers regarding the incompatibility between animal welfare and industrial-intensive breeding.

*The concept of animal welfare does not apply as a regulatory state; it will never be like this. Ultimately, it turns into ensuring specific standards for animals that are not close to their natural state. This means ensuring a standard of living that is not just a complete disaster. (young-occasional consumers)*

*… if you put animals in jail (referred to as industrial breeding), you cannot talk about happiness; it’s as if they put us in jail. Even if you eat well and stay warm, do you feel good in jail? (older-frequent consumer)*

The contrast between industrial farming—defined by consumers as intensive—and non-industrial—understood as domestic and extensive—emerged clearly during the focus groups. The analysis of characteristic words showed that older consumers associated more positive images with extensive-domestic farming than with intensive-industrial farming, as shown in the texts reported.

*When I was a child, rabbits were kept free in the farmyard; later, cages were created, and then industries. (older-frequent consumer)*

*I consider rabbit farms to be among the most dangerous because I imagine them to be similar to battery farms for chickens. Rabbits are raised in cages because they are smaller in size. (young-occasional consumers)*

Older consumers noted that animal welfare measures can be more easily applied to domestically extensive farms, as reported in the following sentences:

*Extensive breeding is how rabbits were raised in the country in the past; the farmers kept them outdoors and free to roam. However, this was not possible at the intensive level. At the farmer’s end, the rabbits had the life of kings. (older-frequent consumer)*

*Considering home breeding, if you close the chicken coop at night and, in the morning, you go to open it, you can see that the hens are happy. They need freedom. (older-frequent consumer)*

Younger-occasional participants also discussed the reasons that encouraged consumers to choose products that come from breeding with animal welfare standards. They agreed that the choice is mainly linked to the protection of consumers’ health, underlining that animals raised according to animal welfare standards are perceived as healthy, and their meat is considered high quality. Some crucial excerpts are as follows:

*If I spend one euro more to buy meat, I do not feel better because I make the animal feel better. I know that I spent more because I bought meat that is better for me. If I eat this meat, I am more comfortable because it is healthier. (young-occasional consumers)*

*Speaking of animal welfare, I wonder what is the purpose for Europe? I think it is beneficial for people. If the animal is bred better, the meat is better, so I eat better, am sick less, and there are fewer costs for health services. (young-occasional consumers)*

### 3.4. Food Safety and Ethical Issues

#### 3.4.1. Survey Results

Ninety percent of the survey respondents (*n* = 1001) stated that there were no health risks associated with the consumption of rabbit meat; 90.3% of those were aged 48 or under, and 89.7% of those aged over 48. The Fisher’s exact test showed that this difference was not statistically significant (*p* = 0.833).

However, 90.2% of the sample somewhat-strongly agreed with the statement that breeding methods influence the safety of the meat we consume, and 56.7% was not at all/slightly agree with the statement that meat comes from industrial farms is safer than that coming from non-industrial farms.

Differences between those aged 48 or under and those aged over 48 were assessed relating to the items reported in Table 6, whose response options were categorized into two categories: ‘somewhat-strongly agree’ and ‘not at all/slightly agree’. Statistically significant differences emerged regarding the following items: ‘Meat from industrial farms is less tasty than meat from non-industrial farms’ (*p* = 0.023), ‘meat from industrial farms is safer than meat from non-industrial farms’ (*p* = 0.030), and ‘only animals raised outdoors live according to animal welfare’ (*p* = 0.001).

Respondents were then asked whether they were willing to buy meat at a slightly higher price if specific circumstances were guaranteed on farms. Table 7 shows that the answer ‘surely yes’ was selected by 70.1% of the respondents concerning measures aimed at reducing the use of antibiotics, while the response ‘yes, but it depends on the final cost’, was more suitable by respondents (46.5%) regarding conditions that guarantee animal welfare. Dependent relationships emerged between the respondents’ age and their willingness to pay for meat at a slightly higher price if the investigated conditions were guaranteed (Table 7). It was found that the final cost is a greater determinant in those aged 48 or under than in those aged over 48.

#### 3.4.2. Focus Group Results

The portions of text concerning the health risk perception of rabbit meat showed different perspectives between younger and older consumers.

In Cluster 5 (25.4% of the classified text), older consumers underlined the relationship between intensive breeding and the use of additives in animal feeds, such as estrogen, medicated feed, and antibiotics:

*In intensive farms, they use a lot of estrogen, vitamins, and medicated feed with medicine in it; otherwise, the animals die. Most rabbit meat in supermarkets came from these farms. (older-frequent consumer)*

*You have to buy it where you think that this rabbit is not medically treated, but raised in a certain way, especially without the use of antibiotics. It is better to prefer farms where antibiotics are not given or, if they are given, as for chickens, I think 15 or 20 days should pass before slaughtering them to ensure the substances are disposed of. (older-frequent consumer)*

However, younger consumers declared that meat from industrial farms was safer than meat from domestic farms. This view originated from consumers’ perception linked to the existence of an efficient control system of the production chains, as reported in the following sentences:

*I feel protected buying industrially raised meat, because there is a health organization that controls and protects us. […] For me, there are more risks coming from outside; it is much worse than what we can find in supermarket meat. (older-frequent consumer)*

*I am more concerned when I eat rabbits from my mother-in-law, who buys it from the neighbor who raises rabbits at home. She claims that these rabbits eat healthy food, but I always say, ‘How do you know if these animals do not drink the water in the little river next to the breeding that could have mercury in it?’ (young-occasional consumers)*

## 4. Discussion

### 4.1. Rabbit Meat Consumption and Animal Welfare Awareness

In this article, data on consumers’ preferences for rabbit meat purchasing and consumption, and on consumers’ perception of rabbit breeding, are reported. These data align with several studies showing consumers’ low consumption of rabbit meat [5,8]. They confirm that consumers eat rabbit meat rarely (at most 1–2 times a year) in Mediterranean countries such as Italy, where there is a long tradition of rabbit meat production and consumption [1,9,26].

The data collected also confirmed that, in Italy, supermarkets were the main place for rabbit meat purchase, followed by butcher stores; in the European context, the large retail chains alternate with farmers’ markets and farms’ points of sale [1,27]. Few respondents declared that they purchased rabbit meat directly from the breeder and street markets. It also emerged that consumers preferred to buy chopped rabbit over a whole carcass or ready-to-eat meat, consistent with other studies [1].

The factors that drive consumers’ purchasing primarily relate to rabbit breeding methods and the appearance of meat. Appearance is often observed in food products purchased from supermarkets and hypermarkets [1]. Following the scheme proposed by Napolitano et al. [28], appearance with odor, flavor, and texture are sensory attributes that can influence consumer acceptance of meat. Visible characteristics of meat (i.e., color) can play a key role in consumers’ choice [8,29], whereas those that cannot be evaluated—even after the normal use of the product (e.g., animal feeding guarantee, environmentally friendly production, respect for animal welfare, etc.)—require supplementary information to be made available to consumers. Information about farming practices can significantly impact consumer expectations, where high animal welfare standards associated with high expected product quality are presented [30]. Animal welfare is recognized as an important component of meat quality assurance and consumer demands, and several studies conducted at the European level confirmed the increase in consumers’ attention toward this aspect [9,31,32,33,34]. The survey showed that breeding with limited use of antibiotics and without animal suffering are the factors that most influence consumers’ purchasing choices regarding rabbit meat. In addition, both quantitative and qualitative data highlight consumers’ different quality perceptions of meat from industrial breeding sold in supermarkets and non-industrial meat purchased directly from farmers.

Among older-frequent consumers, there was a shared perception that rabbit meat raised ‘in the rural/domestic farms’ is the best from an organoleptic perspective, considering it particularly tastier and firmer. This shows that the issue of taste is closely associated with consumers using different breeding methods, emphasizing how the perception of the goodness of meat can change regarding the context of where an animal is raised and the quality of the feed.

Consumers recognized the rural/domestic farms as the only places where animal welfare measures could be applied. This aspect underlines the added value of animal-friendly products, not only concerning organoleptic quality, but also ethical value and food safety. According to other European studies, animal feeding is an important indicator of meat quality and relates to health, particularly among older-frequent consumers [9]. They noticed a relationship between intensive breeding and the use of additives such as estrogens, medicated feeds, and antibiotics in animal feeds, which could affect the safety of meat. Data on animal welfare awareness are in line with a European study [34] aimed at investigating this topic at a general level and not focused only on a specific production chain.

However, a contradiction was observed among the respondents’ views: On the one hand, consumers linked intensive breeding to the production of unsafe meat; on the other hand, they believed that meat purchased at the supermarket was safer than meat from rural/domestic farms, without considering the origin of the meat. This confirms that supermarket standards are synonymous with a guarantee of consumer safety for the purchase of meat [35] and that European consumers have limited knowledge of animal production chains [9]. In general, the data showed that respondents did not perceive rabbit meat as a risky food.

### 4.2. Animal Welfare in Rabbit Breeding

This study underlined the relevance of consumers’ personal health protection. Consumers are willing to pay more for a rabbit meat product originating from breeding, which limits the use of antibiotics and their environmental impact. Consistent with other studies [35], animal welfare is also a crucial aspect of the selection and consumption of animal products. Aligned with another Italian study, younger-occasional participants of focus groups agreed that animals raised with welfare standards are perceived as healthy [36], and their meat is considered of high quality. Respondents defined animal welfare as the absence of stress and animal suffering throughout their life, as well as the possibility of living in an adequate space to ensure the free movement of the animal; they also correlate it to healthy and appropriate feed, confirming data reported in previous Italian studies [36]. There was agreement among all focus group participants regarding the incompatibility between animal welfare and industrial-intensive breeding. Concerning rabbit meat industrial breeding, most consumers indicated small cages as the main breeding method, making it unsuitable for the growth and movement of the rabbit. In addition, they linked the use of cages to a fattening process based on industrial feed and antibiotics. Both survey and focus group respondents did not consider the cage compatible with adequate welfare standards, and this is fully in compliance with the EU resolution, ‘End the Cage Age’ [3]. In general, some respondents were unable to provide a definition of animal welfare; however, around one-third of the respondents did not know how rabbits were raised in intensive farming systems. Other studies have observed that the level of knowledge of consumers on animal welfare is generally low [36,37]. The study also confirmed this regarding rabbit breeding and highlighted the need to increase communication and training activities aimed at operators.

However, the data clearly showed a different perception between industrial farming, defined by consumers as intensive and non-industrial and understood as domestic and extensive. The analysis of the characteristic words of focus group discussions showed that older consumers associated more positive images with extensive-domestic farming rather than intensive-industrial farming. This perception of rabbit farming is not supported by any factual evidence, given that backyard farms also keep animals in small cages, with no specific legal requirement for animal welfare aside from the general prescription of Council Directive 98/58/EC. Older consumers noted that animal welfare measures can be more easily applied to domestically extensive farms. Survey data confirmed that nearly all of the sample agreed with the idea that only animals raised outdoors live according to animal welfare.

Consistent with several studies [5,8,9,27], respondents agreed that, recently, their interest in animal breeding methods has increased, which is also confirmed by the fact that they seek information on rabbit farming methods during purchase. As it is recognized at the European level [27,38], the label is the tool most used by respondents to obtain general information on food products, as well as on how the rabbit was raised (44.1%).

## 5. Conclusions

To encourage the purchase and consumption of rabbit meat, it is important to consider what emerged from the data collected. Consumers of rabbit meat are careful about their health; they actively seek information on how the meat is produced, and they are aware that there are regulations concerning animal welfare in breeding. They also declared that they were willing to buy meat at a slightly higher price, particularly if measures aimed at reducing the use of antibiotics and conditions that guarantee animal welfare are ensured on farms. Aligned with European data, consumers consider animal-friendly meat characteristics as synonymous with quality, underlining the consumers’ positive viewpoint related to breeding methods that focus on the needs of animals. Nonetheless, consumers seemed unaware of the housing systems available for this species.

Starting from these inputs, rabbit meat production sectors should consider the following aspects to accommodate the demands of consumers:

- Promote the development of scientific research on husbandry systems that can ensure the application of animal welfare measures (with particular attention to the absence of stress and suffering);
- Adopt alternative breeding methods using larger rabbit cages suitable for the growth and movement of animals;
- Outline according to public health sector-specific guidelines to ensure good management, biosecurity, vaccination protocols, and health monitoring, which are expected to reduce the need for antimicrobials;
- Enhance the labeling system as a method for communicating fair information to consumers on specific product characteristics.

It is known that the implementation of effective labels and targeted communication strategies represent two crucial tools for the development of marketing efforts aimed at consumers:

- To develop effective marketing communication, it is crucial to consider specific consumers’ knowledge needs:
- Current rabbit breeding methods, explaining the impact they have on rabbits (that is, *why is the cage the main tool used in rabbit breeding?*)
- Scientific knowledge of the animal and its species-specific needs (that is, *can rabbits live together in the same space? What do they need in their lives? etc*.)
- The operation of the control systems of both the farm and all production chains (that is, *How do these controls take place in small, medium, or large farms? How do you guarantee the safety of products?*)

Further investigation would be helpful to compare the data from this study with consumer perceptions related to other meat supply chains and to examine more in-depth the role that other variables (i.e., healthier diets, economic assessments, other ethical drivers) might have on the purchase and consumption of rabbit meat. In recent years, several EU regulations on the welfare of farmed animals have been issued, which is a signal of increasing attention to both animal and consumer needs. The data reported in this article should be considered another useful insight for building a more positive image of the rabbit meat industry.

## Figures and Tables

**Table 1:** Questions of the questionnaire were included in the study.

Sections	Questions	Type of Question (Open-Close)
(1) Rabbit meat consumption and purchase	- How often do you eat rabbit meat?	Close—More options
	- Where do you buy rabbit meat most often?	Close—More options
	- What kind of rabbit format do you usually buy?	Close—More options
	- When you purchase rabbit meat, how could the following factors influence your product choice?	Close—Likert scale (1–10)
	- When you buy rabbit meat, have you ever looked for information on how the animal was raised?	Close—Yes/No
(2) Knowledge and perception of rabbit breeding and animal welfare	- In general, do you think there are regulations that protect animal welfare on a farm?	Close—Yes/No/I don’t know
	- For you, what is the meaning of the expression ‘animal welfare’? Write your definition below.	Open
	- Thinking about animal breeding, which of the following statements do you agree with? (a) The meat from industrial farms is less tasty than meat from non-industrial farms. (b) The industrial breeding facilities are careful to ensure animal welfare. (c) Only animals raised outdoors live according to animal welfare. (d) Breeding methods influence the safety of the meat we consume. (e) The meat from industrial farms is safer than meat from non-industrial farms. (f) In recent years, the consumers’ interest in animal breeding methods has increased.	Close—strongly/somewhat/slightly/not at all agree
	- In general, would you be willing to buy meat at a slightly higher price if the following conditions were guaranteed on farms?	Close—Yes/No
	- How do you think rabbits are raised on industrial farms? Write a short description below.	Open
	- Considering the rabbit industry, is the use of cages in breeding a method compatible with animal welfare?	Close—Yes/No/I don’t know
	- In your opinion, are there health risks associated with the consumption of rabbit meat?	Close—Yes/No
(3) Socio-demographic characteristics of respondents	- What is your gender? - Who do you live with? - What are your educational qualifications? - What is your employment status? - Concerning financial resources, what is your condition at the end of the month?	Close—More options
	- When were you born? - Where do you live? (Specify the city)	Open

**Table 2:** Lexicometric measures of the corpora underwent automatic text analyses.

	Focus Groups	Survey Question (1)	Survey Question (2)
Number of occurrences (Token)	24,844	8480	4975
Number of distinct forms (Type)	3458	1248	976
Type/token ratio	13.9	14.7	19.6
Words that appear only once (Hapax)	54.7	54.4	62.3

**Table 3:** Socio-demographic characteristics of the consumers of rabbit meat (%, *n* = 705).

Characteristics	%
Gender	
Female	48.2
Male	51.8
Where they live in Italy	
Nord-West	26.7
Nord-East	18.7
Centre	20.3
South & Islands	34.3
Age groups	
18–35 years	25.2
36–48 years	26.0
49–60 years	25.4
61–78 years	23.4
Educational qualification	
Primary/lower secondary school	9.8
Professional qualification/higher secondary school diploma	57.7
University diploma/Degree/Post-graduate specialization	32.4
Occupation	
Student	8.2
Looking for his first job	2.3
Homemaker	12.6
Employed	50.8
Unemployed	7.8
Retired	18.3
Financial condition (at the end of the month)	
Very easy	8.8
Quite easy	42.6
With some difficulties	42.0
With many difficulties	6.7
Living with	
Partner & children	33.6
Partner	23.8
Elderly household (with parents)	16.6
Alone	11.9
Only children	11.5
Other	2.6

**Table 4:** Characteristics of the participants were divided into 4 focus groups (*n* = 32).

Group	Type of Consumers	N	Age (Mean)
1	Older—frequent	9	66
2	Older—frequent	11	69
3	Younger—occasional	6	34
4	Younger—occasional	6	39

**Table 5:** Factors that drive consumers’ choice in purchasing rabbit meat. Likert scale 1–10, results of the *t*-tests.

Factors	Total Mean (*n* = 503)	Mean Respondents Aged 48 or under (*n* = 233)	Mean Respondents Aged over 48 (*n* = 270)	*p*-Value
It has been bred with the responsible use of antibiotics	8.09	8.05	8.13	0.662
It looks good	7.92	8.07	7.80	0.099
It has been raised without suffering	7.46	7.42	7.49	0.733
It comes from farms with a low environmental impact	7.35	7.33	7.36	0.904
It is organically farmed	7.21	7.29	7.14	0.459
It was produced close to home	7.04	7.02	7.06	0.816
It can be purchased directly from the breeder	6.91	7.01	6.82	0.378
It is a known brand	5.91	6.11	5.74	0.098
It is economical	5.74	5.88	5.61	0.176

**Table 6:** Considering animal breeding, how much do you agree with the statements below? Results of the Fisher’s exact tests.

Statements	Overall Sample (%, *n* = 1001)	Respondents Aged 48 or under (%, *n* = 504)	Respondents Aged over 48 (%, *n* = 497)	*p*-Value
Farming methods affect the safety of the meat we eat				
Somewhat-strongly agree	90.2	88.7	91.8	0.111
Not at all/slightly agree	9.8	11.3	8.2	
In recent years, consumers’ interest in the way animals are raised has increased				
Somewhat-strongly agree	88.2	87.5	88.9	0.494
Not at all/slightly agree	11.8	12.5	11.1	
Only animals reared in the open air live in animal welfare conditions				
Somewhat-strongly agree	84.1	80.2	88.1	0.001
Not at all/slightly agree	15.9	19.8	11.9	
Meat from industrial farms is less tasty than meat from non-industrial farms				
Somewhat-strongly agree	81.2	78.4	84.1	0.023
Not at all/slightly agree	18.8	21.6	15.9	
Meat from industrial farms is safer than meat from non-industrial farms				
Somewhat-strongly agree	43.3	39.9	46.7	0.030
Not at all/slightly agree	56.7	60.1	53.3	
Industrial livestock farms are careful to protect the animal’s welfare				
Somewhat-strongly agree	30.9	31.3	30.4	0.784
Not at all/slightly agree	69.1	68.7	69.6	

**Table 7:** In general, would you be willing to buy meat at a slightly higher price if the following conditions were guaranteed on farms? (%, *n* = 1001).

Conditions	Overall Sample (%, *n* = 1001)	Respondents Aged 48 or under (%, *n* = 504)	Respondents Aged over 48 (%, *n* = 497)	*p*-Value
Measures to reduce antibiotic use				
Surely yes	70.1	64.9	75.5	0.001
Yes, but it depends on the final cost	26.7	31.0	22.3	
No	3.2	4.1	2.2	
Measures to reduce the environmental impact of livestock farming				
Surely yes	56.7	53.2	60.4	0.049
Yes, but it depends on the final cost	39.2	41.9	36.4	
No	4.1	4.9	3.2	
Measures for the adoption of alternative livestock farming systems over intensive				
Surely yes	55.5	49.4	61.8	0.000
Yes, but it depends on the final cost	39.9	44.8	34.8	
No	4.6	5.8	3.4	
Conditions protecting the animal welfare				
Surely yes	50.4	45.8	55.1	0.013
Yes, but it depends on the final cost	46.5	50.8	42.1	
No	3.1	3.4	2.8	

**Figure A1:**
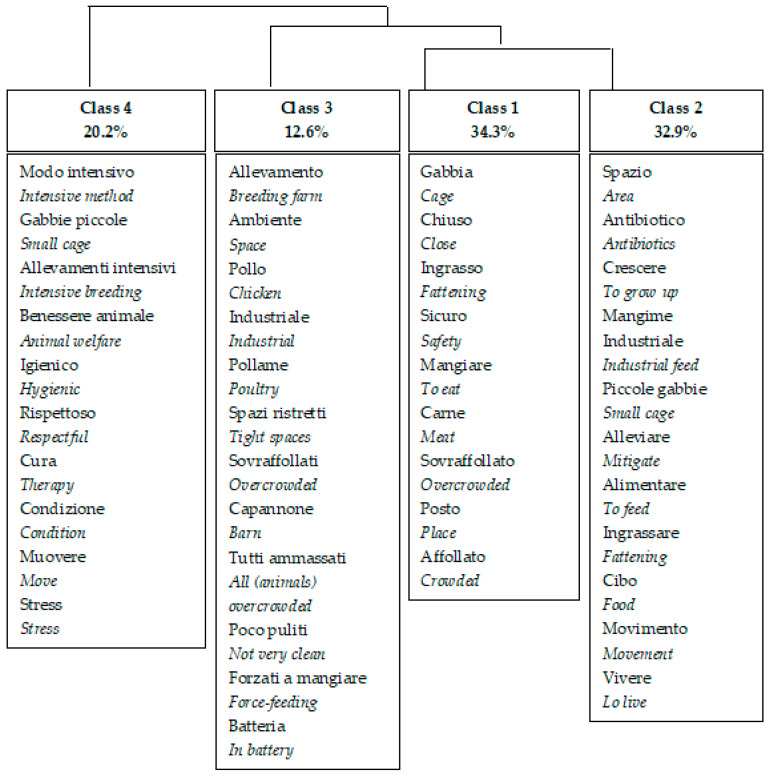
The word translation of the 4 clusters as reported in Figure 5.

**Figure A2:**
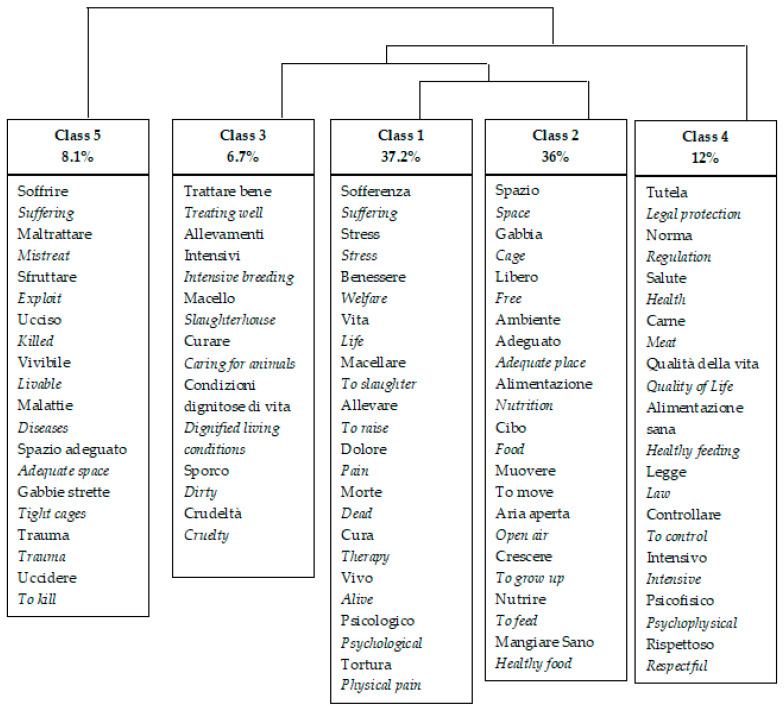
The word translation of the 5 clusters as reported in Figure 6.

## Data Availability

The data that support the findings of this study are available from the corresponding author upon reasonable request.
